# Additive value of texture analysis based on breast MRI for distinguishing between benign and malignant non-mass enhancement in premenopausal women

**DOI:** 10.1186/s12880-021-00571-x

**Published:** 2021-03-12

**Authors:** Yu Tan, Hui Mai, Zhiqing Huang, Li Zhang, Chengwei Li, Songxin Wu, Huang Huang, Wen Tang, Yongxi Liu, Kuiming Jiang

**Affiliations:** 1 Department of Radiology, Guangdong Women and Children Hospital, No.521, Xingnan Road, Panyu District, Guangzhou, 511400 China; 2 Department of Radiology, The Third Affiliated Hospital of Guangzhou Medical University, Guangzhou, China

**Keywords:** Breast, MRI, Non-mass enhancement, Texture analysis, Additive value

## Abstract

**Background:**

Non-mass enhancement (NME) is a diagnostic dilemma and highly reliant on the experience of the radiologists. Texture analysis (TA) could serve as an objective method to quantify lesion characteristics. However, it remains unclear what role TA plays in a predictive model based on routine MRI characteristics. The purpose of this study was to explore the value of TA in distinguishing between benign and malignant NME in premenopausal women.

**Methods:**

Women in whom NME was histologically proven (n = 147) were enrolled (benign: 58; malignant: 89) was retrospective. Then, 102 and 45 patients were classified as the training and validation groups, respectively. Scanning sequences included Fat-suppressed T2-weighted and fat-suppressed contrast-enhanced T1-weighted which were acquired on a 1.5T MRI system. Clinical and routine MR characteristics (CRMC) were evaluated by two radiologists according to the Breast Imaging and Reporting and Data system (2013). Texture features were extracted from all post-contrast sequences in the training group. The combination model was built and then assessed in the validation group. Pearson’s chi-square test and Mann–Whitney U test were used to compare categorical variables and continuous variables, respectively. Logistic regression analysis and receiver operating characteristic curve were employed to assess the diagnostic performance of CRMC, TA, and their combination model in NME diagnosis.

**Results:**

The combination model showed superior diagnostic performance in differentiating between benign and malignant NME compared to that of CRMC or TA alone (AUC, 0.887 vs 0.832 vs 0.74). Moreover, compared to CRMC, the model showed high specificity (72.5% vs 80%). The results obtained in the validation group confirmed the model was promising.

**Conclusions:**

With the combined use of TA and CRMC could afford an improved diagnostic performance in differentiating between benign and malignant NME.

**Supplementary Information:**

The online version contains supplementary material available at 10.1186/s12880-021-00571-x.

## Background

According to the Breast Imaging-Reporting and Data System (BI-RADS) magnetic resonance imaging (MRI) lexicon (2013), non-mass enhancement (NME) is defined as a special MRI enhancement mode, which is different from the surrounding enhanced breast parenchyma. It has no space occupation effect and typically contains scattered adipose and glandular tissues [1, 2]. NME is often encountered on MRI screening. It might appear in benign breast lesions, such as focal adenosis or fibrocystic and inflammatory changes and can also manifest in malignant lesions, such as lobular carcinoma, diffuse invasive breast cancer, invasive ductal carcinoma, ductal carcinoma in situ (DCIS), and occasionally, some special types of breast cancers [3–5].

Distinguishing between benign and malignant NME is a challenge in breast MRI-based diagnosis. Since biopsy guided by MRI is not popular, over or delayed surgery is frequent [6, 7]. Previous studies have shown that specific morphological MRI features and kinetic curve patterns of NME could offer some guidance [8, 9]. However, in daily clinical practice, the use of these methods is considered rather limited and controversial. Recent studies have claimed that MRI feature reports have significant inter-observer variability and a lack of quantitative indicators and repeatability [10]. Moreover, breast tissue affected by hormone effects would add to the difficulty of diagnosis of NME, especially in premenopausal women [11].

Texture analysis (TA) uses a computer-assisted approach to analyze the statistical difference in the grey-level pixel intensity in extracted medical images, thereby providing an objective way to quantify tumor characteristics and growth patterns [12, 13], which is not feasible in traditional radiology evaluations. TA based on breast MRI has shown great potential in terms of offering molecular biology information. It has been used as a “digital biopsy” to distinguish between malignant tumor and benign lesions [14], to predict outcomes for patients [14], and to assess treatment responses [15]. For lesions presenting as NME, it remains unclear what role TA plays in a predictive model based on routine MRI characteristics. The purpose of this study, therefore, was to explore the value of TA in distinguishing between benign and malignant NME in premenopausal women.

## Methods

### Study population

**Fig. 1:**
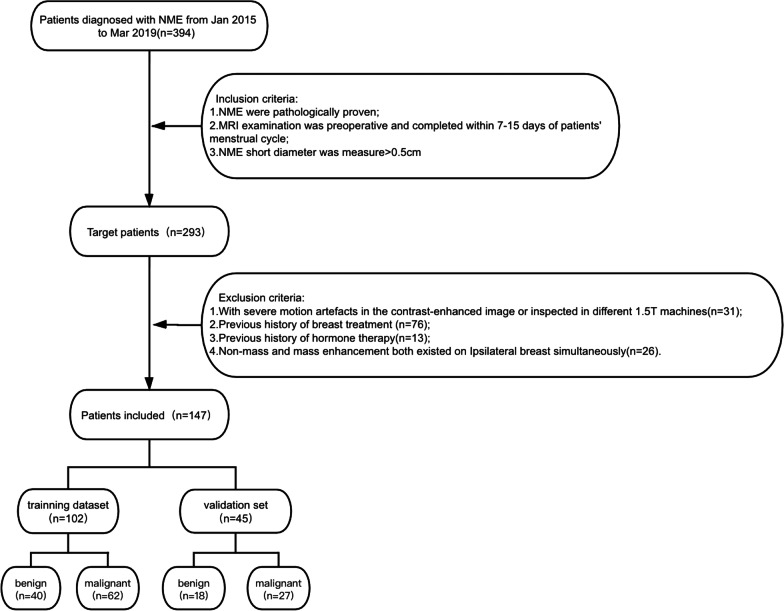
The workflow of the inclusion and exclusion criteria of this study

We searched all breast MRI examination reports in our radiology information systems from January 2015 to March 2019 and selected “NME” as the retrieval keyword. MRI data for 394 female patients were found. Two hundred and ninety-three of the patients met the following criteria: (1) NME confirmed by pathological analysis; (2) MRI performed within 1 week before the surgery and during 7–15 days of the patients’ menstrual cycle to decrease the false-positive results provided by background enhancement (BPE) [16]; and (3) define the shortest part of its measuring diameter is greater than 0.5 cm, such that the possible adverse effects on the texture features extracted from DICOM data were minimized. The exclusion criteria were as follows: (1) severe motion artifacts in the contrast-enhanced images or the use of different 1.5T machines for scanning (n = 31); (2) a history of treatment for breast cancer, i.e., surgery, biopsy, radiotherapy, or chemotherapy (n = 76); (3) a history of hormone therapy (n = 13); and (4) NME and mass enhancement both existed on the ipsilateral breast simultaneously (n = 26). Finally, 147 patients were included in this retrospective study. Among these patients, 58 had benign lesions while 89 had malignant lesions. Figure 1 shows a flow chart of the inclusion and exclusion criteria for this study. Table 1 summarizes the histological types of these two groups of lesions.

**Table 1:** Histological types of lesions in the two groups: benign and malignant non-mass enhancement

Tumor group	Number (cases)	Percentage (%)
Benign non-mass enhancement	58	39.5
Fibrocystic changes	33	22.4
Inflammation	20	13.6
Mix	6	4.1
Malignant non mass enhancement	89	60.5
Invasive ductal carcinoma	16	10.9
Atypical ductal hyperplasia	18	12.2
Ductal carcinoma in situ	11	7.5
Invasive ductal carcinoma	13	8.8
Invasive micropapillary carcinoma	3	2
Apocrine carcinoma	1	0.7
Mix	26	17.7

### MRI protocol

All MRI studies were conducted using 1.5T (T) dedicated breast MRI system (Aurora Imaging Technology, North Andover, MA), equipped with an integrated breast-specific coil. The patients were scanned in the prone position. Dynamic enhanced imaging included a total of five phases performed using a T1-weighted fat-suppression sequence in the axial plane with TR = 29 ms, TE = 4.8 ms, flip angle = 45°, FOV = 36 × 36 cm, slice thickness = 1.12 mm, and gap = 0. A total of 160 slices were used to cover the entire breast. After acquiring one set of pre-contrast images, the contrast medium (gadobenate diethylenetriamine pentaacetic acid, Gd-DTPA, Magnevist) was administered as a bolus injection (infusion rate: 2 ml/s; dose: 0.2 mmol/kg per patient weight), followed by flushing with 20 ml of normal saline. Both the contrast medium and normal saline were injected into the vein through an automated contrast delivery system (Sonic Shot GX; Nemoto Kyorindo, Japan). Four sets of post-contrast enhanced images were obtained. The acquisition interval time for each was 3 min. In addition, a fat-suppressed T2-weighted sequence was performed with the following parameters: TR = 6680 ms, TE = 5.3 ms, matrix size = 320 × 192, FOV = 36 cm, slice thickness = 3 mm, and gap = 0.

All images were further analyzed in using a dedicated workstation equipped with computer-aided detection for further analysis. The time-intensity curve (TIC) was generated by analyzing different color codes of fluid and edema.

### Clinical and routine MRI characteristic assessment

Clinical and routine MRI characteristics (CRMC) were used to distinguish between benign and malignant NME. The clinical variable assessed was age. Routine MRI characteristics were visually assessed by two breast radiologists (reader 1, with over 10 years of experience; reader 2, with more than 13 years of experience) separately and independently. Both readers had access to the patients’ previous clinical and/or imaging information, except for the histopathological diagnosis, during their initial reading. The NME evaluation involved a comparison of both breasts to avoid false-positive results caused by BPE. The final diagnosis was based on the consensus between the two radiologists.

**Fig. 2:**
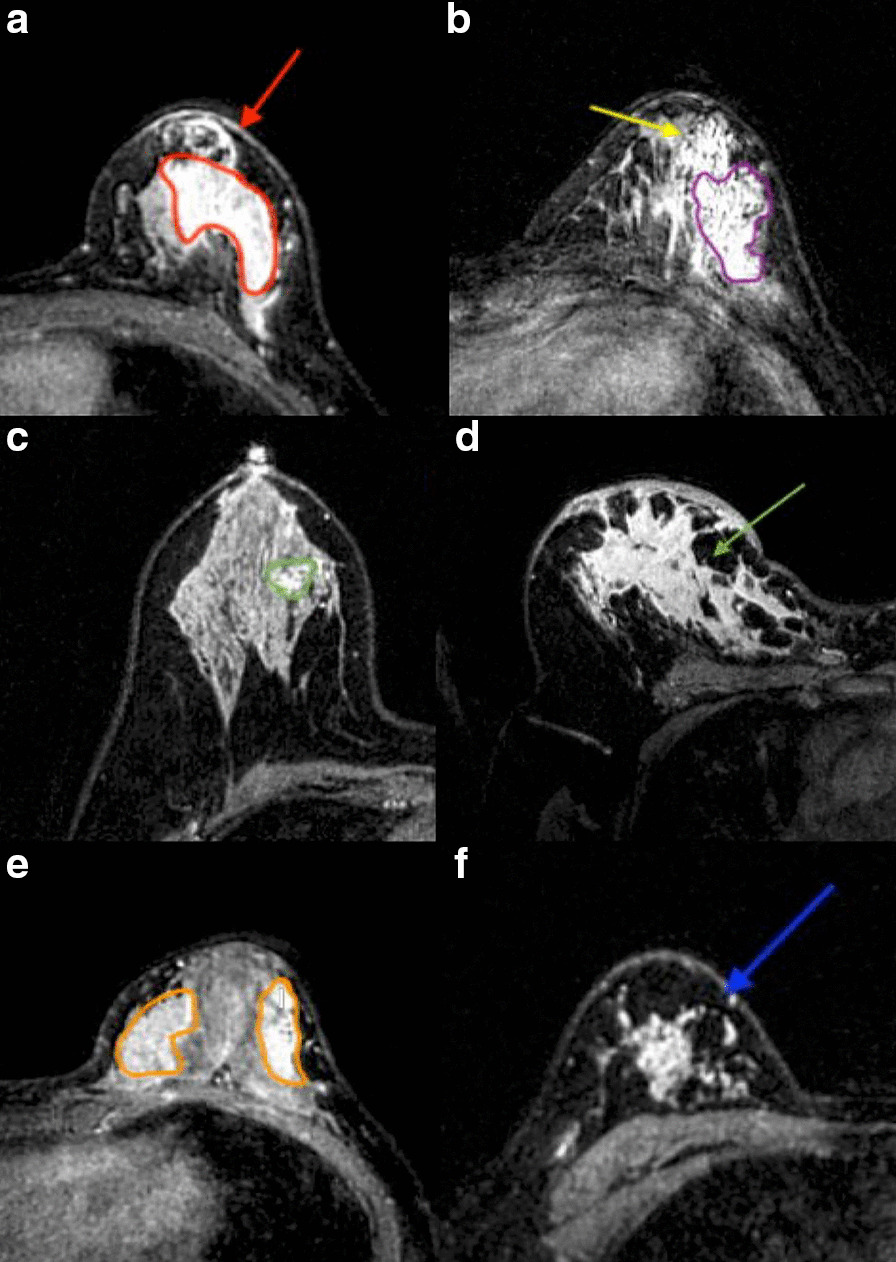
The sequence of dynamic imaging performed using axial T1-weighted fat-suppression. **a** The second contrast-phase image shows an NME with homogeneous internal enhancement patterns (red irregular shape) and a linear distribution (red arrow). **b** The third contrast-phase image shows that the entire lesion enhancement was heterogeneous, with intermingling local, cluster-ring, and clumped enhancement (purple irregular shapes) and the appearance of segmental distribution (yellow arrow). **c**, **d** respectively show the focal (green irregular shape) and diffuse (green arrow) distribution patterns of NME in the fifth contrast-phase image. **e**, **f** Respectively show the regional (orange irregular shape) and multiple-region (blue arrow) distributions in the second contrast phase

The selection of image characteristics was based on the BI-RADS-MRI (2013) diagnostic guidelines, including lesion distribution pattern (focal/linear/segment/regional/multiple regions/diffuse) appeared or not and internal enhanced mode (homogeneous/heterogeneous/clumped/clustered ring) presence or absence. Figure 2 shown the MRI examples of these features. The relationship between lesion signal intensity and time was evaluated by TIC, which was categorized into persistent, plateau, and washout patterns [2].

### TA

The 2nd to 5th contrast sequences were input into Mazda 4.6 (a public software developed by the Institute of Electronics in Lodz Technical University, Poland) for TA [17–19]. For each case, a region of interest (ROI) was manually delineated by reader 1. The slice of the 2nd contrast phase that was selected to draw the ROI met the following criteria: (1) slice showing the largest cross-section area of the NME and with no visible necrotic areas; and (2) selection of the largest slice when multiple lesions were found on one slice of the same breast. Then, reader 2 double-checked the ROI setting. If there was a disagreement on the boundary, the readers resolved it by discussing between themselves. The same ROI was placed on the same slice for the 3rd to the 5th contrast phases to ensure that the ROI could accurately reflect the change of NME grey-scale intensity in different post-contrast sequences caused by drug metabolism. In addition, to decrease the impact of image brightness and contrast variation on the TA results, the grey-level intensity was normalized within μ + 3σ (μ, mean grey value; σ, mean standard deviation [20].

**Fig. 3:**
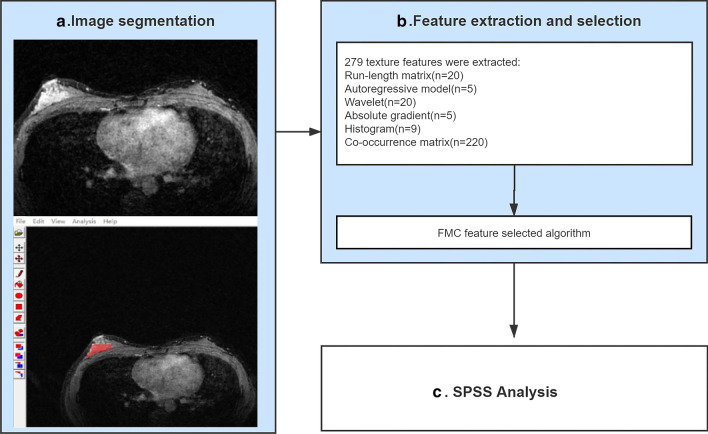
Workflow for identification of benign and malignant non-mass enhancement based on texture analysis. Processes in blue boxes were performed in MaZda

The MaZda TA report could offer almost 300 texture parameters for each ROI. There are six texture feature categories included in this analysis: run-length matrix (RLM), autoregressive model (ARM), wavelet, absolute gradient (GrM), histogram, and the co-occurrence matrix parameters (COM). Additionally, for each ROI, the RLM algorithm was computed in the vertical, horizontal, 45-degree, and 135-degree directions, i.e., four times in all. The COM algorithm was derived from four directions (θ = 0, 45, 90, and 135), and the distance of the pixels ranged from 1 to 5, i.e., for each ROI, each of the five distances was counted separately in the four directions, making up a total of 20. As shown in Table 2 [17], the combined use of the feature extraction algorithms, including the Fisher coefficient, mutual information, classification error probability, and average correlation coefficients (POE + ACC), afforded the screening of the top 30 texture features with the strongest ability to distinguish between benign and malignant NME. The TA workflow chart for NME is shown in Fig. 3.

**Table 2:** Texture parameters computed by MaZda

Texture feature algorithm	Parameters
RLM	Grey-level/run-length nonuniformity, long/short run emphasis, fraction of image in runs
ARM	Model parameter vector includes 4 parameters; Sigma: standard deviation of the noise
Wavelet	Energy of the wavelet coefficients in subbands
GrM	Kurtosis, skewness, variance, mean, percentage of pixels with a nonzero gradient
Histogram	Skewness; mean; kurtosis; variance; and perc. 01%, perc. 10%, perc. 50%, perc. 90%, and perc. 99%
COM	Angular second moment, correlation, contrast, sum of squares, inverse difference moment, sum variance, sum average, sum entropy, entropy, difference variance, difference entropy

RLM, run-length matrix; ARM, Auto-regressive model; GrM, absolute gradient; COM, co-occurrence matrix parameters

### Statistical analysis

Mann–Whitney U test was used to compare continuous variables. Categorical variables evaluated by Pearson’s chi-square test (n > 40, TRC > 5) or Yates’s correction for continuity (n > 40, 1 ≤ TRC < 5). Univariate logistic regression was performed initially on each variable, and the variables showing statistical significance in the univariate logistic regression were further analyzed using multiple logistic regression to establish a discriminating model.

For assessing the diagnostic efficacy of each approach, the receiver operating characteristic (ROC) and the area under the curve (AUC) were evaluated. All data analyses were performed on SPSS 22.0(Windows version), and a P value less than 0.05 was considered statistically significant.

### Validation study

To evaluate the diagnostic performance of the combined model, the data were divided into a training dataset of 102 cases and a validation set of 45 cases by simple random sampling with an approximate method in SPSS 22.0. The ratio of the two was 7:3. The mean ages of the training and validation cohorts were 38.7 + 6.8 and 38.1 + 8.7 years, respectively. The number of cases of benign and malignant NME in the training data set were 40 and 62, respectively; the corresponding numbers for the validation set were 18 and 27. The holdout cross-validation method was used to verify the diagnostic performance of the discriminating model constructed in multivariate logistic regression. AUROC values were applied as a measure of success. *p* < 0.05 was considered statistically different.

## Results

### CRMC

Among the 102 cases with pathologically proven NME, 40 cases showed benign findings, and 62 cases showed malignancy. Patient age in cases showing benign findings (36.1 ± 6.8) was lower than that in the cases showing malignancy (40.4 ± 6.2). The difference between the two groups was statistically significant (*p* < 0.001).

With the respect to the conventional MRI features of NME, a linear, multiple-region distribution and the washout time-intensity pattern were significantly more frequent in malignant lesions, whereas a distribution of focal areas and a plateau time-intensity pattern were common findings in benign lesions (*p* < 0.05). In contrast to the distribution (regional, segmental, diffuse) and internal enhancement modes, the persistence time-intensity patterns of NME did not differ significantly between benign and malignant NME (*p* > 0.05) (Table 3).

**Table 3:** Statistical results of clinical and routine MRI findings

Clinical and routine MR characteristics	Benign (n = 40)	Malignant (n = 62)	*p* value
Age (years)	36.1 ± 6.8 (22–49)	40.4 ± 6.2 (25–54)	< 0.001*
Distribution			
Focal area	9 (22.5%)	5 (8.1%)	0.039*
Linear	10 (25%)	41 (66.1%)	< 0.001*
Segment	33 (82.5%)	56 (90.3%)	0.247
Regional	23 (57.5%)	26 (41.9%)	0.125
Multiple regions	12 (30.0%)	32 (51.6%)	0.031*
Diffuse	3 (7.5%)	4 (6.5%)	0.838
Internal enhancement			
Homogeneous	9 (22.5%)	13 (20.1%)	0.854
Heterogeneous	31 (77.5%)	49 (79.1%)	0.854
Clumped	32 (80.0%)	52 (83.9%)	0.617
Clustered ring	9 (22.5%)	21 (33.9%)	0.218
TIC pattern			
Persistent	16 (40%)	22 (35.5%)	0.645
Plateau	13 (32.5%)	6 (9.7%)	0.004*
Washout	11 (27.5%)	34 (54.8%)	0.007*

^*^p value, statistically significant (*p* < 0.05)

Multivariate logistic regression analysis of CRMC showed 3 independent indicators with statistical significance to discriminate benign and malignant NME, namely, age, linear distribution, and multiple-region distribution (*p* < 0.05). For ROC analysis, the AUC was 83.7% (CI, 0.76–0.91) and standard error was 0.04 (*p* < 0.001). The sensitivity was 80.6% and the specificity was 72.5%.

### Texture features

One, four, and eight statistically significant texture features were selected from 2nd, 3rd, and 5th contrast phases respectively, and no statistically significant texture features were found in the 4th contrast phase (Table 4). Multivariate logistic regression analysis of TA found that three statistically significant texture features could discriminate benign and malignant NME, which were as follows: S (5, 5) Correlate (*p* = 0.01) from the second contrast phase, Perc.90% (*p* = 0.002), and S (4, − 4) Correlate (*p* = 0.001) from the fifth contrast phase. For ROC analysis, the AUC was 74% (CI, 0.64–0.84) and standard error was 0.05, (*p* < 0.001). The sensitivity was 64.5% and the specificity was 70%.

**Table 4:** Statistically significant texture features in the FMC method of contrast phases

Dynamic enhanced phases	Texture parameters	Z value	*p* value	Algorithm model
2nd phase	S (5,5) Correlat^a^	− 2.467	0.01	COM
3rd phase	Perc.99%	− 2.20	0.03	Histogram
	Mean	− 2.32	0.02	Histogram
	Perc.50%	− 2.28	0.02	Histogram
	Perc.90%	− 2.40	0.02	Histogram
5th phase	Perc.99%	− 2.29	0.02	Histogram
	Perc.90%^a^	− 2.55	0.01	Histogram
	Perc.50%	− 2.31	0.02	Histogram
	Mean	− 2.31	0.02	Histogram
	Teta 3	− 2.05	0.04	ARM
	S (4, − 4) Correlat^a^	− 2.41	0.02	COM
	S (5, − 5) Correlat	− 2.51	0.01	COM
	Variance	− 2.02	0.04	GRM

FMC, methods included Fisher coefficient, mutual information, classification error probability, and average correlation coefficients algorithms;

^a^Data, the statistically significant texture features in the multiple regression analysis, which would be input into the combined diagnosis model to distinguish between benign and malignant NME

### Combined model

Multiple logistic regression was used to create a combined model to predict malignant NME by using age, linear, multiple regions distribution, Perc.90%, S (5, 5) Correlate and S (4, − 4) Correlate, which were statistically significant and independent factors (*p* < 0.05) (Table 5).

**Table 5:** Logistic regression results of identifying benign and malignant NME in the training dataset

CRMC and texture features	B value	*p* value	Odds ratio	95% confidence level
Age^a^	0.162	< 0.01	1.176	1.07–1.292
Multiple regions of distribution^a^	1.431	0.015	4.181	1.313–13.311
Linear^a^	2.283	< 0.01	9.81	2.959–32.521
S(5,5)Correlat^b^	0.697	0.034	2.009	1.053–3.832
S(4, − 4)Correlat^c^	− 0.92	< 0.01	0.399	0.199–0.797
Perc. 90%^c^	− 0.612	0.044	0.542	0.299–0.984

^a^Data, features of CRMC

^b^Data, texture feature from the 2nd contrast phase

^c^Data, texture features from the 5th contrast phase

**Fig. 4:**
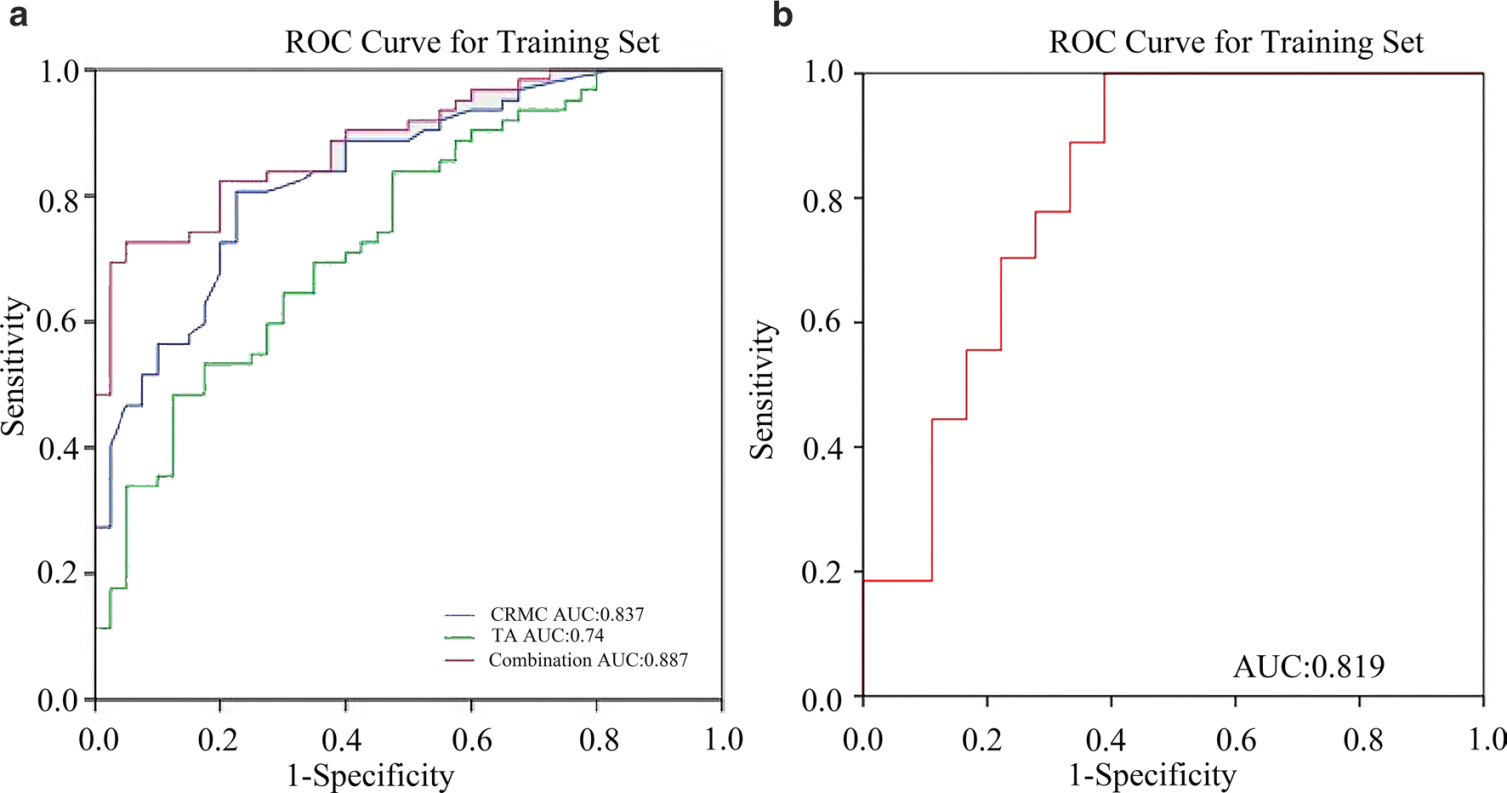
The ROC curves in different diagnostic methods to distinguish benign and malignant NME. **a** CRMC, TA, and combination diagnostic models in the training dataset; **b** the combination model in the validation data set

For discriminate benign and malignant NME, the combined model shown the best diagnostic efficiency, in comparison to the efficiencies of CRMC and TA alone. Its AUC was 88.7% (CI 0.83–0.95) and standard error was 0.03 (*p* < 0.001). The sensitivity was 82.3% and specificity was 80% (Table 6, Fig. 4)**.**

**Table 6:** ROC results for CRMC, TA, and combination model

	AUC (95% CI)	Sensitivity (%)	Specificity (%)	*p* value
CRMC	83.7% (0.76, 0.91)	80.6	72.5	< 0.01
TA	74% (0.64, 0.84)	64.5	70	< 0.01
Combine				
Training set	88.7% (0.83, 0.95)	82.3	80	< 0.01
Validation set	81.9% (0.68, 0.96)	77.8	72.2	< 0.01

### Validation study results

The validation set included 18 benign and 27 malignant cases of NME, with a mean patient age of 38.1 ± 8.7 years (range, 16 to 52 years; *p* < 0.001). To verify the repeatability of the combined model constructed by multiple logistic regression, the holdout cross-validation method was used. Its AUROC was 81.9% (CI 0.68–0.92), sensitivity was 77.8%, and specificity was 72.2%, as shown in Table 6.

## Discussion

In this study, we assessed the diagnostic value of texture features in discriminating benign and malignant NME. To this end, we compared three diagnostic methods: models using TA or CRMC alone and a model using a combination of these. The diagnostic efficacy obtained with TA alone was not significantly higher than that with CRMC (74% vs 83.2%), but their combination resulted in additive effects and improved diagnostic performance (AUC = 0.887, *p* < 0.05) (Table 6, Fig. 4). At the same time, the combined model was successfully verified as a promising diagnostic model in the validation set (AUC = 0.819). Our results also indicated that reducing the influence of BPE could improve the diagnostic specificity of CRMC, and this study yielded more information about the use of TA for assessment of NME in premenopausal women.

The morphological features and dynamic contrast enhanced (DCE) parameters in benign and malignant NME have been studied extensively. Many investigators confirmed the results obtained by Tozaki et al. for the NME internal enhancement and distribution patterns, and they suggested that most benign NMEs appeared with a linear distribution and homogeneous internal enhancement, whereas lesions exhibiting a heterogeneous and clustered ring internal enhancement with segmental distribution were highly suggestive of malignant NME [21–23]. For the time-intensity curve (TIC), previous studies have demonstrated no statistically significant differences between benign and malignant lesions in any type of enhancement pattern [24]. However, the results of the present study were inconsistent with these findings, since the present study showed that a linear, segmental, and multiple-regions enhancement distribution and washout kinetic pattern were detected more frequently in malignancy, whereas a focal area-enhanced distribution with a plateau kinetic curve pattern was more likely to appear in benign lesions. Moreover, this study showed no evidence that a clumped, cluster ring with a homogeneous or heterogeneous structure was statistically significant in identifying benign or malignant status. For ROC analysis, Shao et al. performed a meta-analysis of diagnostic performance based on morphological characteristics and enhanced parameters by using pooled weighted estimates, and their results indicated low sensitivity (50%) and high (80%) specificity. In contrast, the results of this study indicated high sensitivity (80.6%), while the specificity was not high (72.5%) [25].

The discrepancy might be attributed to the following reasons. First, the inclusion criteria were different. The criteria for this study included measures to reduce the interference of BPE in NME diagnosis. Since some investigators believed that when BPE manifests as asymmetric, regional, or focal distribution, it was difficult to distinguish BPE from NME [26–28]. Moreover, BPE might interfere with the delineation of tumor boundaries [29]. However, this major factor that affected the diagnostic accuracy of NME in premenopausal women was ignored by previous studies; Second, the interpretation of morphologic features in MR images was highly dependent on the radiologist’s experience level and lacked reproducibility. This might account for the different sensitivities and specificities of NME diagnosis with routine MRI features.

At present, texture analysis by extracting the features of the particular area in an image is considered to be a repeatable and efficient auxiliary diagnostic method, the principle of which is based on the spatial distribution of the intensity level in each pixel [10, 13]. Unfortunately, to the best of our knowledge, few studies used TA in NME. Newell et al. first used TA to diagnose NME, and their ROAUC was not high (0.76) [30]. The results of subsequent studies were similar, and our TA results were no exception, with the AUC, sensitivity, and specificity all lower than those with CRMC, indicating that the diagnostic efficiency of TA alone in NME diagnosis was not high. Some investigators had used TA combined with breast MRI morphology features to distinguish between phyllodes and fibroadenomas tumors, while others had combined TA with DWI parameters to predict the response to neoadjuvant chemotherapy for breast cancer, and their results demonstrated that combined TA could improve the diagnostic performance [31, 32]. On the basis of previous studies, we tried to use the combination of TA and CRMC in NME diagnosis. Our results showed that the diagnostic performance of the model combining TA and CRMC was greater than that achieved with CRMC or TA alone (AUC: 0.887 vs 0.832 vs 0.74). Furthermore, in comparison with CRMC, the combined model also showed greater specificity (72.5% vs 80%).

In addition, this study found that features from the 2nd and 5th contrast sequences were more meaningful in discriminating benign and malignant NME, which was consistent with previous results showing that the time to enhancement (TTE) and maximum slope (MS) in DCE-MRI could distinguish benign and malignant NME. The pathological and pharmacokinetic mechanisms differed in benign and malignant lesions. Malignant tumors had abundant vascularity and highly permeable vessel walls that allowed easier transfer of the contrast agent from vessels to the extravascular space was easier; thus, malignant lesions had shorter TTE and larger MS, while the benign lesions showed the opposite findings [33, 34]. This could explain why in the combined model, the texture features extracted from the 2nd and 5th contrast sequences were independently relevant to discriminate benign and malignant NME.

### Limitations

The limitations of our studies should be noted: First, we used a small-sized retrospective database, which is subject to potential bias. Further studies using larger datasets and validating the combined model on other equipment should be attempted in the future. Moreover, manual ROI segmentation led to inevitable measurement errors; thus, the next step is to develop artificial intelligence tools that can accurately recognize these lesions.

## Conclusions

The addition of TA to CRMC could improve the diagnostic performance in NME, providing a noninvasive quantitative approach for NME diagnosis that could distinguish malignant and benign lesions and decrease the excessive surgery or benign NME core needle biopsy (Additional file 1).

## Supplementary Information

**Additional file 1**. Flow-chart shows the MRI scanning plan for this study. Process in blue-box is T1WI pre-contrast sequence and process in the pink-box is T1WI-pro-contrast sequence

## Data Availability

The datasets used and/or analyzed during the current study are available from the corresponding author on reasonable request.

## Abbreviations

MRI: Magnetic resonance imaging
NME: Non-mass enhancement
TA: Texture analysis
CRMC: Clinical and routine MR characteristics
BI-RADS: Breast imaging-reporting and data system
BPE: Background enhancement
TIC: Time-intensity curve
ROI: Region of interest
AUC: Area under the curve
DCE-MRI: Dynamic contrast-enhanced magnetic resonance imaging
TTE: Time to enhancement
MS: Maximum slope

## References

[1] Mercado CL (2014). BI-RADS update. Radiol Clin North Am.

[2] Edwards SD, Lipson JA, Ikeda DM, Lee JM (2013). Updates and revisions to the BI-RADS magnetic resonance imaging lexicon. Magn Reson Imaging Clin N Am.

[3] Giess CS, Raza S, Birdwell RL (2013). Patterns of nonmasslike enhancement at screening breast MR imaging of high-risk premenopausal women. Radiographics.

[4] Milosevic ZC, Nadrljanski MM, Milovanovic ZM, Gusic NZ, Vucicevic SS, Radulovic OS (2017). Breast dynamic contrast enhanced MRI: fibrocystic changes presenting as a non-mass enhancement mimicking malignancy. Radiol Oncol.

[5] Chadashvili T, Ghosh E, Fein-Zachary V, Mehta TS, Venkataraman S, Dialani V, Slanetz PJ (2015). Nonmass enhancement on breast MRI: review of patterns with radiologic-pathologic correlation and discussion of management. AJR Am J Roentgenol.

[6] Santoso MR, Yang PC (2016). Magnetic nanoparticles for targeting and imaging of stem cells in myocardial infarction. Stem Cells Int.

[7] Dratwa C, Jalaguier-Coudray A, Thomassin-Piana J, Gonin J, Chopier J, Antoine M, Trop I, Darai E, Thomassin-Naggara I (2016). Breast MR biopsy: pathological and radiological correlation. Eur Radiol.

[8] Gity M, Ghazi Moghadam K, Jalali AH, Shakiba M (2014). Association of different MRI BIRADS descriptors with malignancy in non mass-like breast lesions. Iran Red Crescent Med J.

[9] Sakamoto N, Tozaki M, Higa K, Tsunoda Y, Ogawa T, Abe S, Ozaki S, Sakamoto M, Tsuruhara T, Kawano N (2008). Categorization of non-mass-like breast lesions detected by MRI. Breast Cancer.

[10] Pinker K, Chin J, Melsaether AN, Morris EA, Moy L (2018). Precision medicine and radiogenomics in breast cancer: new approaches toward diagnosis and treatment. Radiology.

[11] Giess CS, Yeh ED, Raza S, Birdwell RL (2014). Background parenchymal enhancement at breast MR imaging: normal patterns, diagnostic challenges, and potential for false-positive and false-negative interpretation. Radiographics.

[12] Marino MA, Pinker K, Leithner D, Sung J, Avendano D, Morris EA, Jochelson M (2020). Contrast-enhanced mammography and radiomics analysis for noninvasive breast cancer characterization: initial results. Mol Imaging Biol.

[13] Holli K, Laaperi AL, Harrison L, Luukkaala T, Toivonen T, Ryymin P, Dastidar P, Soimakallio S, Eskola H (2010). Characterization of breast cancer types by texture analysis of magnetic resonance images. Acad Radiol.

[14] Chitalia RD, Kontos D (2019). Role of texture analysis in breast MRI as a cancer biomarker: a review. J Magn Reson Imaging.

[15] Fan M, Wu G, Cheng H, Zhang J, Shao G, Li L (2017). Radiomic analysis of DCE-MRI for prediction of response to neoadjuvant chemotherapy in breast cancer patients. Eur J Radiol.

[16] Kajihara M, Goto M, Hirayama Y, Okunishi S, Kaoku S, Konishi E, Shinkura N (2013). Effect of the menstrual cycle on background parenchymal enhancement in breast MR imaging. Magn Reson Med Sci.

[17] Szczypinski PM, Strzelecki M, Materka A, Klepaczko A (2009). MaZda—a software package for image texture analysis. Comput Methods Programs Biomed.

[18] Materka A (2004). Texture analysis methodologies for magnetic resonance imaging. Dialogues Clin Neurosci.

[19] Castellano G, Bonilha L, Li LM, Cendes F (2004). Texture analysis of medical images. Clin Radiol.

[20] Waugh SA, Purdie CA, Jordan LB, Vinnicombe S, Lerski RA, Martin P, Thompson AM (2016). Magnetic resonance imaging texture analysis classification of primary breast cancer. Eur Radiol.

[21] Tozaki M, Fukuda K (2006). High-spatial-resolution MRI of non-masslike breast lesions: interpretation model based on BI-RADS MRI descriptors. AJR Am J Roentgenol.

[22] Chen QL, Luo Z, Zheng JL, Li XD, Liu CX, Zhao YH, Gong Y (2012). Protective effects of calcium on copper toxicity in *Pelteobagrus fulvidraco*: copper accumulation, enzymatic activities, histology. Ecotoxicol Environ Saf.

[23] Chikarmane SA, Michaels AY, Giess CS (2017). Revisiting nonmass enhancement in breast MRI: analysis of outcomes and follow-up using the updated BI-RADS atlas. AJR Am J Roentgenol.

[24] El Khouli RH, Macura KJ, Jacobs MA, Khalil TH, Kamel IR, Dwyer A, Bluemke DA (2009). Dynamic contrast-enhanced MRI of the breast: quantitative method for kinetic curve type assessment. AJR Am J Roentgenol.

[25] Shao Z, Wang H, Li X, Liu P, Zhang S, Cao S (2013). Morphological distribution and internal enhancement architecture of contrast-enhanced magnetic resonance imaging in the diagnosis of non-mass-like breast lesions: a meta-analysis. Breast J.

[26] Hegenscheid K, Schmidt CO, Seipel R, Laqua R, Ohlinger R, Hosten N, Puls R (2012). Contrast enhancement kinetics of normal breast parenchyma in dynamic MR mammography: effects of menopausal status, oral contraceptives, and postmenopausal hormone therapy. Eur Radiol.

[27] DeMartini WB, Liu F, Peacock S, Eby PR, Gutierrez RL, Lehman CD (2012). Background parenchymal enhancement on breast MRI: impact on diagnostic performance. AJR Am J Roentgenol.

[28] Brooks JD, Sung JS, Pike MC, Orlow I, Stanczyk FZ, Bernstein JL, Morris EA (2018). MRI background parenchymal enhancement, breast density and serum hormones in postmenopausal women. Int J Cancer.

[29] Amano Y, Woo J, Amano M, Yanagisawa F, Yamamoto H, Tani M (2017). MRI texture analysis of background parenchymal enhancement of the breast. Biomed Res Int.

[30] Newell D, Nie K, Chen JH, Hsu CC, Yu HJ, Nalcioglu O, Su MY (2010). Selection of diagnostic features on breast MRI to differentiate between malignant and benign lesions using computer-aided diagnosis: differences in lesions presenting as mass and non-mass-like enhancement. Eur Radiol.

[31] Mai H, Mao Y, Dong T, Tan Y, Huang X, Wu S, Huang S, Zhong X, Qiu Y, Luo L (2019). The utility of texture analysis based on breast magnetic resonance imaging in differentiating phyllodes tumors from fibroadenomas. Front Oncol.

[32] Eun NL, Kang D, Son EJ, Park JS, Youk JH, Kim JA, Gweon HM (2020). Texture analysis with 30-T MRI for association of response to neoadjuvant chemotherapy in breast cancer. Radiology.

[33] Goto M, Sakai K, Yokota H, Kiba M, Yoshida M, Imai H, Weiland E, Yokota I, Yamada K (2019). Diagnostic performance of initial enhancement analysis using ultra-fast dynamic contrast-enhanced MRI for breast lesions. Eur Radiol.

[34] Yang X, Dong M, Li S, Chai R, Zhang Z, Li N, Zhang L (2020). Diffusion-weighted imaging or dynamic contrast-enhanced curve: a retrospective analysis of contrast-enhanced magnetic resonance imaging-based differential diagnoses of benign and malignant breast lesions. Eur Radiol.

