# Supplementary figures and images for: Additive value of texture analysis based on breast MRI for distinguishing between benign and malignant non-mass enhancement in premenopausal women
Source: BMC Med Imaging. 2021 Mar 12;21:48. doi: 10.1186/s12880-021-00571-x (PMC7953679; doi:10.1186/s12880-021-00571-x)

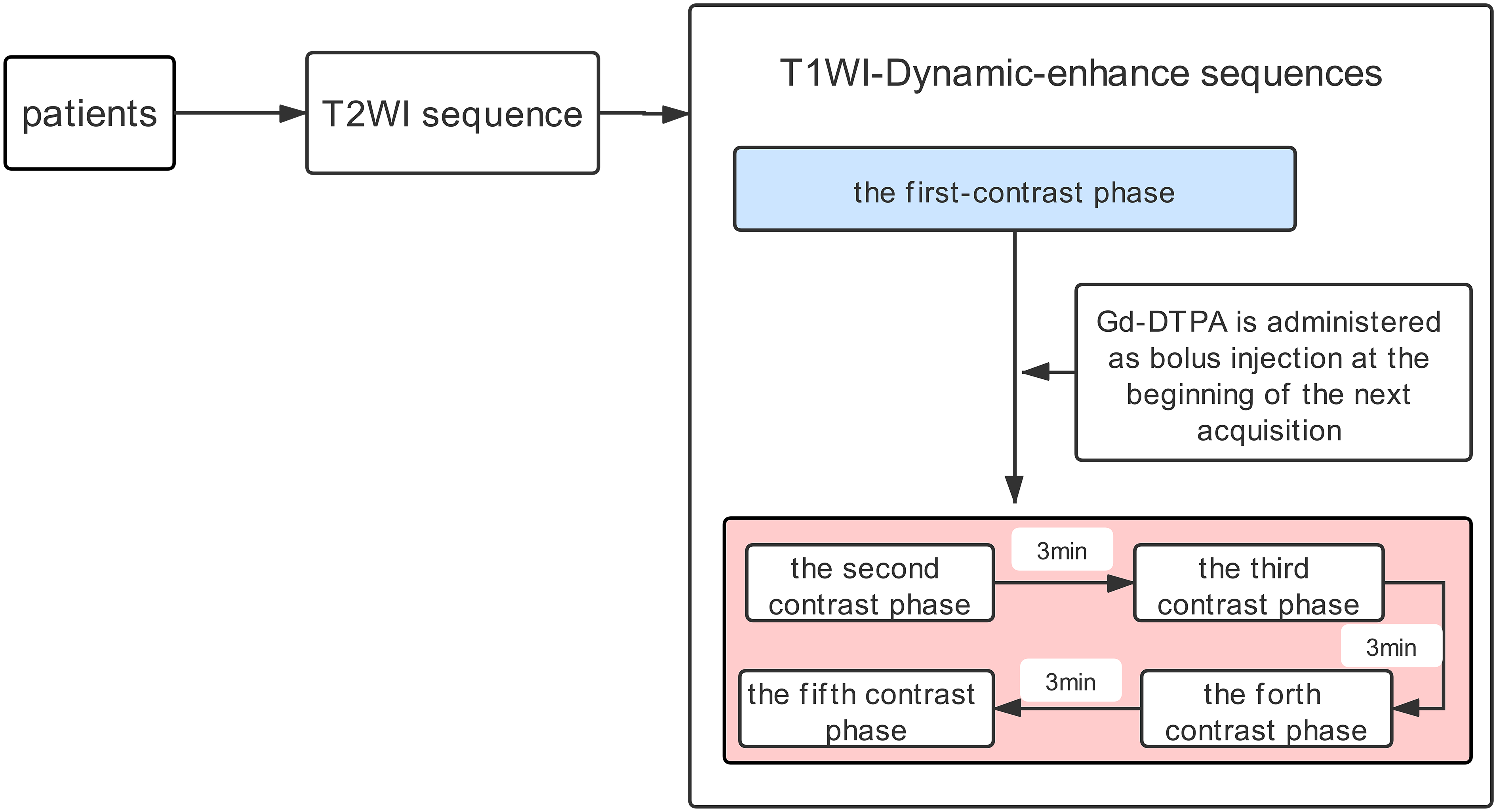

Supplement: Supplementary file 1 — Additional file 1. Flow-chart shows the MRI scanning plan for this study. Process in blue-box is T1WI pre-contrast sequence and process in the pink-box is T1WI-pro-contrast sequence [file 12880_2021_571_MOESM1_ESM.tif]
